# Redesigning a Pediatric Resuscitation Course Using a Hybrid Model: A Program Evaluation

**DOI:** 10.7759/cureus.110042

**Published:** 2026-06-01

**Authors:** Hadas Katz-Dana, Hadas Yechiam, Tzvia Shelter, Ehud Rosenbloom

**Affiliations:** 1 Pediatric Emergency Medicine, Meir Medical Center, Kfar Saba, ISR

**Keywords:** advanced pediatric life support (apls), flipped classroom, pediatrics, resuscitation, simulation-based education

## Abstract

Introduction: Traditional advanced pediatric life support (APLS) courses require two full days of in-person instruction, creating challenges related to time, cost, and faculty resources. Flipped-classroom models may improve efficiency while maintaining educational outcomes. This study evaluated a hybrid APLS course combining interactive asynchronous learning with a simulation-focused in-person day.

Methods: We conducted a mixed-methods program evaluation at a pediatric emergency department. Participants completed five interactive asynchronous modules followed by a one-day, simulation-based course. Outcomes included performance on the standardized APLS examination and post-course evaluations using Likert-scale items and open-ended questions. Results from the hybrid course were descriptively compared with aggregated data from the traditional two-day format.

Results: Thirty-eight clinicians participated. All completed the course and passed the APLS examination (100% pass rate), with scores descriptively similar to institutional benchmarks from the traditional format. Learner satisfaction was high, with mean ratings ranging from 4.92 to 5.00 (five-point scale). Asynchronous modules and simulation activities received the highest ratings. Descriptive comparisons showed similar learner evaluation scores across domains. Qualitative analysis identified themes of improved preparedness, high engagement, and strong preference for the hybrid model. Course duration was reduced by 50%, and participant cost decreased by 25%.

Discussion: A hybrid, flipped-classroom APLS model incorporating interactive asynchronous learning and simulation-based training was feasible and well-received. Educational outcomes were descriptively consistent with those observed in the traditional format while improving efficiency and reducing resource requirements. This approach may support scalable delivery of pediatric resuscitation training.

## Introduction

Advanced pediatric life support (APLS) courses are central to preparing clinicians to recognize and manage critically ill children. These courses teach essential cognitive knowledge, critical procedural, and teamwork skills necessary for high-stakes pediatric resuscitation [1-3]. Traditionally, APLS training is delivered over two intensive, contiguous days. This traditional format combines extensive didactic lectures with skill stations and simulation-based scenarios [4]. While effective in achieving learning outcomes, this model is inherently time- and resource-intensive, requiring substantial faculty availability, dedicated training space, and, critically, the removal of participant clinicians from clinical duties for an extended period. These logistical and financial demands pose a significant barrier to maintaining continuous, high-quality training, particularly for healthcare systems operating with tight educational budgets or managing staff shortages in busy emergency and intensive care settings [5-7].

Over the past decade, the flipped-classroom model has emerged as an effective strategy in health professions education. In this approach, learners engage with the foundational didactic content asynchronously (e.g., via pre-recorded modules or readings) before the course [8-10]. This strategy aligns strongly with principles of adult learning theory, promoting self-directed study and ensuring learners arrive prepared for higher order application. This allows the in-person time to focus exclusively on interactive learning activities, problem-solving, and experiential training [11]. Multiple studies have demonstrated that flipped formats can maintain or improve knowledge outcomes and enhance learner engagement, particularly when paired with high-fidelity simulation-based instruction [8,12-14]. In resuscitation education, flipped models are non-inferior to traditional teaching, but with improved efficiency [11].

However, in reported implementations, the asynchronous component of flipped courses relies primarily on passive content delivery, such as recorded lectures or reading materials. Less attention has been given to flipped designs that emphasize active, interactive learning during the asynchronous phase, particularly through digital platforms that require learner decision-making, case progression, and immediate feedback. Evidence supporting the application of such active, technology-enhanced flipped models within pediatric resuscitation training remains limited.

In addition, few studies in pediatric resuscitation education have evaluated a model in which the entire in-person component is dedicated exclusively to hands-on skills and simulation, following completion of asynchronous preparatory learning. Demonstrating that such a model can preserve educational outcomes while reducing course duration, faculty burden, and participant cost is essential for institutions seeking scalable and sustainable training solutions.

In response to these gaps, we implemented a redesigned APLS course utilizing a flipped-classroom model in which all didactic content was delivered through interactive asynchronous modules, and the in-person day was devoted entirely to skills practice and simulation-based training. The primary objective of this study was to evaluate the feasibility, educational outcomes, and operational benefits of this new format. We hypothesized that the flipped-classroom, simulation-focused model would be feasible, well accepted by participants, associated with satisfactory immediate knowledge performance, and operationally more efficient than the traditional format in terms of time and cost.

## Materials and methods

Study design and setting

This was a single-center educational program evaluation of a redesigned APLS course using a flipped-classroom, simulation-focused format. This was a mixed-methods program evaluation using a convergent design employing quantitative assessment of immediate cognitive knowledge and learner satisfaction, complemented by qualitative analysis of open-ended feedback. Learner evaluations from the hybrid course were compared descriptively with aggregated evaluation data from the traditional two-day APLS course delivered at the same institution before the redesign. Because historical learner-level data from the traditional format were not available for matched or paired analysis, inferential statistical testing was not performed.

The study was conducted at a large pediatric emergency department in Israel with approximately 43,000 annual visits. APLS courses at this institution are delivered as part of continuous professional development (CPD) for physicians and nurses in pediatric emergency medicine, pediatrics, anesthesia, and prehospital care.

Participants

A convenience sample of 38 healthcare professionals, representing all participants enrolled in the first two consecutive iterations of the hybrid APLS course between 2024 and 2025, was included in the evaluation. The cohort included a diverse professional mix, including pediatric emergency physicians, general pediatricians, anesthesiologists, and senior nurses. Inclusion criteria required active clinical involvement in pediatric resuscitation; all participants who completed the full course intervention were included in the analysis. The cohort comprised 20 pediatric medicine residents, nine pediatric ward nurses, five pediatric emergency department nurses, two pediatric day care unit nurses, one anesthesia resident, and one orthopedic surgery resident.

Curriculum development and content alignment

The traditional APLS course at our institution consisted of two consecutive days (approximately 16 total hours), including didactic lectures, skill stations, and simulation scenarios. In the redesigned hybrid model, the course was restructured to a single eight-hour in-person day, representing a 50% reduction in contact time.

The hybrid course was developed through a structured curriculum mapping process to ensure alignment with the original APLS course. All learning objectives from the traditional two-day course were identified and mapped to corresponding components of the redesigned format, including asynchronous modules, skill stations, and simulation scenarios.

Content experts in pediatric emergency medicine reviewed all modules to ensure comprehensive coverage of key domains. The asynchronous modules were designed to deliver equivalent cognitive content, while the in-person component focused on application through hands-on skills practice and simulation-based scenarios.

All didactic lectures were replaced by five interactive online modules created and delivered through the Affinity Learning platform (Affinity Learning, Canada). The modules are institutional educational materials developed for this course; they are not publicly accessible and are distributed only to registered course participants after enrollment. Each module was approximately 20 minutes in duration and included narrated slides, embedded knowledge checks, branching clinical cases, and animations. Selected modules included interactive components such as simulated clinical dialogues and guided decision-making exercises. These components provided learners with immediate automated feedback during module completion, supporting reinforcement of clinical reasoning and decision-making. Following the modules, learners were required to complete a 30-question pre-course test. The course director monitored participant activity through the platform to ensure full completion of all five modules and successful passage of the pre-course test (minimum passing score of 70% required) before learners were eligible to attend the in-person day, ensuring prerequisite knowledge acquisition.

The in-person component eliminated all didactic lectures and was focused exclusively on active, experiential learning, including structured, hands-on skill stations, high-fidelity simulation scenarios, structured debriefings led by trained instructors, and case-based discussions emphasizing critical decision-making.

The project was reviewed by the Institutional Review Board (IRB) and determined to meet criteria for exemption from formal review as a program-evaluation initiative, consistent with institutional guidelines. All participants provided implied consent by voluntarily completing the course and associated evaluation instruments.

Outcome measures

Knowledge Assessment

Learners completed the standardized APLS multiple-choice examination at the end of the in-person day. This is the same knowledge test used in the traditional two-day format. Scores were recorded as the percentage of correct responses and evaluated against the institutional pass threshold (70%). Results were contextually compared to historical institutional benchmark pass rates.

Learner Satisfaction

Participants completed a post-course evaluation that included standardized Likert-scale items (1 = strongly disagree to 5 = strongly agree) assessing satisfaction with key domains, including perceived preparedness from asynchronous modules, quality of the simulation experience, and overall instructional effectiveness, as well as open-ended questions assessing the strengths of the hybrid format and suggestions for improvement.

Cost and Efficiency Metrics

Operational efficiency was quantified by documenting the reduction in total course duration, required faculty teaching hours, and participant fees, comparing the hybrid model descriptively against the previous traditional format.

Data analysis

Quantitative data were summarized using descriptive statistics, including means, standard deviations, medians, and proportions. Learner evaluation outcomes from the hybrid course were descriptively compared with aggregated evaluation data from the traditional two-day APLS course delivered at the same institution. Because cohorts were independent and historical data were not available at the individual participant level for matched analysis, comparisons were descriptive rather than statistically significant. Qualitative comments were analyzed using thematic analysis to identify recurring patterns and themes. All analyses were performed using standard spreadsheet and statistical software.

## Results

Thirty-eight clinicians participated in the hybrid APLS courses, including pediatric emergency physicians, general pediatricians, anesthesiologists, and senior nurses. All participants completed the asynchronous affinity modules in advance and attended the full simulation day.

Knowledge outcomes

All participants completed the standardized APLS multiple-choice examination. Scores were descriptively similar to institutional benchmarks from the previous two-day format, with a 100% pass rate. Individual examination scores were not retained in program records; only pass/fail outcomes were documented.

Learner Satisfaction

Quantitative evaluations demonstrated uniformly high satisfaction with the hybrid model. Mean scores on five-point Likert scales ranged from 4.92 to 5.00, with minimal variability (SD: 0-0.26). Simulation activities received the highest possible ratings, and the asynchronous modules were also rated highly.

**Figure 1:**
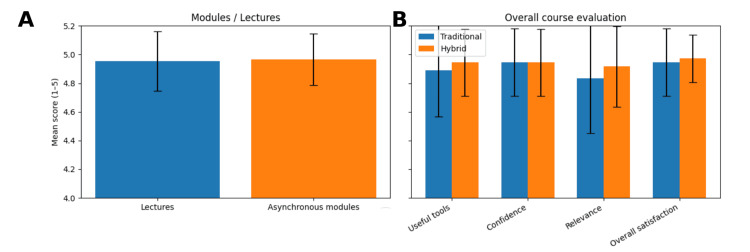
Comparison of learner evaluation scores between traditional and hybrid APLS course formats Mean learner evaluation scores on a 1–5 Likert scale with standard deviation error bars for the traditional two-day APLS course (blue) and the hybrid course (orange). (A) Comparison of ratings for traditional didactic lectures and the corresponding asynchronous modules in the hybrid format. (B) Comparison of ratings across overall course-evaluation domains. APLS: Traditional advanced pediatric life support.

Comparative analysis of learner evaluations between the hybrid and traditional course formats demonstrated similar ratings across all domains. Scores for the asynchronous modules in the hybrid format were descriptively similar to ratings for lectures in the traditional course (4.98 ± 0.15 vs. 4.96 ± 0.21). Simulation activities were rated highly in both formats, with slightly higher scores in the hybrid course (5.00 ± 0.00 vs. 4.97 ± 0.17). Ratings for overall usefulness, relevance to clinical practice, and overall satisfaction were maintained or modestly improved, whereas confidence scores remained unchanged (Figure 1​​​​​).

Mann-Whitney U tests demonstrated no statistically significant differences between the hybrid and traditional formats across the evaluated domains (all p > 0.25), supporting the comparability of the hybrid format (Table 1).

**Table 1:** Comparison of learner evaluation scores between traditional and hybrid APLS courses Values are presented as mean ± SD. Between-group comparisons were performed using the Mann-Whitney U test. U values are reported as the smaller Mann-Whitney U statistic, and p-values are two-tailed. APLS: Traditional advanced pediatric life support.

Domain	Traditional, N = 36, Mean ± SD	Hybrid, N = 38, Mean ± SD	Mann-Whitney U Test	P-Value
Modules/Lectures	4.95 ± 0.19	4.93 ± 0.23	654	0.500
Simulation activities	4.97 ± 0.12	4.99 ± 0.08	664	0.537
Useful tools	4.89 ± 0.32	4.95 ± 0.23	644	0.366
Confidence	4.94 ± 0.23	4.95 ± 0.23	682	0.967
Relevance	4.83 ± 0.38	4.92 ± 0.27	624	0.256
Overall satisfaction	4.94 ± 0.23	4.97 ± 0.16	664	0.537

Qualitative Findings

Analysis of open-ended responses revealed three central themes. Open-ended comments were collected from all 38 participants. Thematic coding was performed independently by two coders, and themes were derived inductively from the comment data.

Enhanced Preparedness and Flexibility

Participants reported that asynchronous modules enabled self-paced review and improved readiness for simulation.

“The modules were excellent, I could review the material whenever I needed.”

High Engagement During Simulation-Based Learning

Learners valued dedicating the entire in-person day to hands-on skills and complex scenarios.

“Focusing the whole day on simulation made the learning much more effective.”

Strong Preference for the Hybrid Model

Many participants expressed enthusiasm for expanding this format to additional courses.

“This is how all courses should be taught.”

Cost and operational efficiency

The hybrid model reduced the course duration from two full days to one day, reduced faculty teaching hours, and decreased participant fees by 25% (1,800₪ to 1,400₪). Reduced reliance on lecture space increased scheduling flexibility and improved sustainability, with no observed deterioration in immediate knowledge performance or learner satisfaction. Table 2 presents a structured comparison of the traditional and hybrid course formats across key operational parameters.

**Table 2:** Operational comparison of the traditional two-day and hybrid one-day APLS course formats. APLS: Traditional advanced pediatric life support; MCQ: Multiple-choice question; ₪: Israeli new shekel.

Parameters	Traditional Format (2-Day)	Hybrid Format (1-Day)
In-person course days	2 days	1 day
Total in-person active hours	16 hours	8 hours
Asynchronous pre-course learning	None	5 modules (~100–150 min) + pre-course MCQ
Didactic lectures (in-person)	Yes (sick child assessment, shock, respiratory, cardiac, metabolic, and neurological emergencies)	Replaced by asynchronous modules
Faculty/staff (Day 1)	2	Not required (asynchronous)
Faculty/staff (Day 2/course day)	6	6
Rooms/venues required	3 conference rooms (both days)	3 rooms (1 day only)
Participant cost	1,800₪	1,400₪ (−22%)

## Discussion

This study evaluated a flipped-classroom, simulation-focused redesign of the APLS course at an urban pediatric emergency center. The hybrid model was feasible, well-received, and descriptively consistent with the traditional two-day format across measured outcomes. Learners achieved descriptively similar knowledge outcomes, reported uniformly high satisfaction, and expressed a clear preference for the redesigned structure. Importantly, these outcomes were maintained despite a substantial reduction in in-person instructional time.

Comparative analysis of learner evaluations showed that transitioning from traditional lectures to asynchronous modules did not negatively affect perceived educational quality. Ratings for core content delivery, simulation activities, and overall course evaluation were stable or slightly improved in the hybrid format. Learners’ self-reported confidence in managing pediatric emergencies remained unchanged across formats, a finding consistent with preliminary program-evaluation data suggesting no observed deterioration in immediate outcomes. Statistical comparison of overall learner satisfaction between formats did not demonstrate a significant difference, further supporting the comparability of the two approaches.

The high immediate pass rates and learner satisfaction observed in this study are consistent with prior literature reporting descriptively similar outcomes for flipped-classroom approaches in resuscitation education [15-18]. However, most published implementations rely primarily on passive transfer of didactic content through recorded lectures or static online materials. In contrast, the hybrid component of the present intervention emphasized active, interactive learning during the asynchronous phase, incorporating case-based decision-making exercises, simulated clinical encounters, and automated feedback to support clinical reasoning. This design was intended to foster clinical reasoning and cognitive readiness for high-fidelity simulation rather than simply shifting traditional lectures to a digital format.

By relocating didactic content to asynchronous modules, the hybrid model allowed the in-person APLS day to focus exclusively on experiential learning, including hands-on skills training and high-fidelity simulation. Learners’ qualitative feedback emphasized improved preparedness and deeper engagement, reflecting core principles of adult learning theory [19].

The operational advantages observed in this study are particularly relevant for resource-constrained healthcare systems. Few published studies have described the cost or efficiency outcomes of innovations in resuscitation training, making these findings an important contribution [16,20]. The ability to deliver high-quality APLS instruction with reduced time, space, and staffing requirements suggests a scalable and sustainable model for pediatric emergency education.

Limitations

This single-center pilot study involved a relatively small cohort, consistent with early implementation. The evaluation focused on immediate knowledge and satisfaction outcomes; future studies should assess skill performance, knowledge retention, and clinical impact over time. Although statistical comparisons were performed for selected outcomes, the study was not powered for formal noninferiority testing because of the nonrandomized design, lack of matched participants, and strong ceiling effects in evaluation scores. In addition, comparisons were based on independent cohorts with aggregated historical data, which limits the ability to draw definitive conclusions regarding equivalence. Accordingly, the findings should be interpreted as supporting comparability and feasibility rather than definitive equivalence between formats.

Additionally, individual APLS examination scores were not retained in program records; only pass/fail outcomes were documented. This precluded reporting of score distributions or formal numerical comparisons with historical benchmarks. Future program evaluations should incorporate a systematic collection of granular assessment data. Furthermore, the absence of historical benchmark score distributions limits the ability to make meaningful numerical comparisons of knowledge performance across cohorts, and stronger claims about knowledge comparability will require access to individual-level data from both formats.

## Conclusions

A hybrid redesign of APLS training was feasible and well-received, with immediate outcomes descriptively similar to the traditional format while reducing in-person time and participant cost. Further research is needed to evaluate skill performance, knowledge retention, clinical transfer, and broader implementation.
